# Body Image Satisfaction, Overweight Dissatisfaction, and Exercise Persistence: A Self-Determination Theory Approach

**DOI:** 10.3390/bs16020208

**Published:** 2026-01-31

**Authors:** Rogério Salvador, Lucio Naranjo, Ruth Jiménez-Castuera, Ricardo Rebelo-Gonçalves, Diogo Monteiro

**Affiliations:** 1 Research Center in Sport Sciences, Health Sciences and Human Development (CIDESD), 6201-001 Covilhã, Portugal; 2 School of Education and Social Sciences (ESECS), Polytechnic University of Leiria, 2411-901 Leiria, Portugal; 3 CIEQV–Life Quality Research Centre, Polytechnic University of Leiria, 2411-901 Leiria, Portugal; 4 Faculty of Sport Sciences, University of Extremadura, 10003 Cáceres, Spain; ruthji@unex.es; 5 Interdisciplinary Center for the Study of Human Performance (CIPER), Faculty of Sport Science and Physical Education, University of Coimbra, 3040-248 Coimbra, Portugal

**Keywords:** body image perception, interpersonal behavior, basic psychological needs, exercise persistence, self-determination, exercise practitioners

## Abstract

Grounded in Self-Determination Theory (SDT), this study tested the hypothesis that body image perception delineates distinct motivational pathways, linking the perceived interpersonal style of exercise professionals to basic psychological needs, motivation quality, and long-term exercise persistence intentions. A sample of 821 regular exercisers was divided into two groups based on body image: “Satisfied” (n = 276) and “Dissatisfied due to Overweight” (n = 545). Participants completed validated measures of perceived interpersonal behaviors (supportive/thwarting), basic psychological need satisfaction/frustration, motivational regulation, and exercise persistence intention. A clear divergent pattern emerged, strongly supporting the main hypothesis. The “Satisfied” group reported a positive pathway: perceiving more need-supportive behaviors from instructors was associated with greater satisfaction of autonomy, competence, and relatedness, which in turn correlated with more self-determined motivation and stronger persistence intentions. Conversely, the “Dissatisfied” group reported a negative pathway: perceiving more need-thwarting behaviors was associated with greater need frustration, which correlated with more non-self-determined motivation and weaker persistence intentions. Measurement invariance confirmed these pathways are comparable across groups. The findings highlight that body image perception is a key correlate of distinct motivational experiences in exercise settings. Crucially, they underscore the significant association between the professional’s perceived interpersonal style and these pathways. Fostering need-supportive environments that enhance autonomy, competence, and relatedness is associated with more adaptive motivation and adherence, offering a valuable framework for practitioners aiming to support clients, particularly those with body image concerns.

## 1. Introduction

### 1.1. Research Background and Problem Statement

The high prevalence of physical inactivity represents a significant global public health challenge, associated with increased morbidity and diminished well-being (Lee et al., 2012; Guthold et al., 2018, 2020). In Portugal, this issue is stark, with 73% of the population not engaging in physical exercise, where a lack of motivation or interest is a primary barrier (European Commission, 2022). This reality underscores the critical need to identify the psychological determinants of long-term exercise persistence. While motivation is a well-established key factor (Edmunds et al., 2008; Rodrigues et al., 2019b), its quality and origins require deeper investigation, particularly in relation to potent self-perceptions like body image (Salvador et al., 2023).

### 1.2. Theoretical Framework and Key Constructs

Self-Determination Theory (SDT) (Ryan & Deci, 2000) provides the foundational framework for this study, positing that the satisfaction of basic psychological needs (BPNs) for autonomy, competence, and relatedness is essential for fostering high-quality, self-determined motivation, which in turn leads to lasting behavioral persistence (Hagger & Chatzisarantis, 2008; Rodrigues et al., 2018). The social environment, particularly the interpersonal style of exercise professionals, is a primary driver of these need-based experiences, with supportive behaviors fostering positive outcomes and thwarting behaviors leading to motivational deficits and dropout (Rodrigues et al., 2018, 2020a, 2020b; Yan et al., 2024).

A critical, yet less understood, factor in this motivational sequence is the individual’s body image. Body image perception is a powerful influence on behavior and mental health. Negative perceptions are linked to psychological distress and maladaptive behaviors, whereas positive perceptions can facilitate health-promoting activities like exercise (Cash, 2004; Salvador et al., 2023; Sarwer et al., 2005). From an SDT perspective, body image can be understood as a significant antecedent that shapes the individual’s experience of the social context and their basic psychological needs (Ryan & Deci, 2017). Specifically, dissatisfaction with one’s body, particularly when linked to overweight perceptions, can directly contribute to need frustration (Ntoumanis et al., 2021). It can thwart the need for competence by fostering a sense of physical inadequacy (Brunet et al., 2012, undermine autonomy by predisposing individuals to internalize external pressures as controlling reasons for behavior (Tylka & Homan, 2015), and hinder relatedness by increasing social physique anxiety (Brunet & Sabiston, 2009). This state of need frustration is the proposed mechanism that fosters non-self-determined motivation, where exercise is regulated by guilt, shame, or contingent self-worth (Tylka & Homan, 2015; Panão & Carraça, 2020; Yan et al., 2024). Conversely, a positive body image, aligned with a more secure and integrated self-concept, is theorized to facilitate greater receptivity to need-supportive interactions, thereby promoting need satisfaction and more self-determined motivation (Avalos et al., 2005).

### 1.3. Research Gap and Necessity of the Study

Emerging evidence suggests that body image dissatisfaction may predispose individuals to a more non-self-determined of motivation (Panão & Carraça, 2020; Tylka & Homan, 2015), while a positive body image may align more readily with self-determined motivation. This creates a complex interplay where body image may shape how individuals perceive the behaviors of their instructors, which subsequently influences their psychological need satisfaction and type of motivation (Salvador et al., 2023).

However, a critical gap remains. While previous research has established the importance of instructor behaviors and BPN for motivation (Deci et al., 2017; Rodrigues et al., 2020a, 2020b) and others have noted the link between motivation type and body image (Panão & Carraça, 2020), the literature lacks an integrated test of the full causal sequence. It remains unclear whether and how body image satisfaction and overweight dissatisfaction act as key antecedents, setting individuals on divergent motivational pathways that ultimately determine their intention to persist with exercise.

Furthermore, Self-Determination Theory proposes that the motivational sequence linking interpersonal context, basic psychological needs, and behavioral regulation represents a universal psychological process (Ryan & Deci, 2000, 2017). To rigorously test whether this proposition holds across different body image perceptions, and to ensure that any observed differences between groups are attributable to genuine divergences in psychological experiences rather than to measurement artifacts, it is essential to test the structural invariance of the proposed motivational model across groups (Deci & Ryan, 2000; Vansteenkiste et al., 2020). This examination is grounded in the SDT premise of universal psychological processes (Ryan & Deci, 2017).

### 1.4. Research Questions

To address this gap, this study employs SDT to examine a comprehensive model. The central research questions are as follows:

How do body image satisfaction and overweight dissatisfaction influence the perception of interpersonal behaviors, the satisfaction and frustration of basic psychological needs, motivational regulation, and, ultimately, exercise persistence?

Does the proposed motivational model function in a structurally equivalent manner across individuals satisfied with their body image and those dissatisfied due to being overweight?

### 1.5. Literature Review and Hypothesis Development

To test this model, we propose the following core hypothesis and the specific sequential paths:

**H1.**  *Individuals satisfied with their body image and individuals dissatisfied due to being overweight will report significantly different profiles across the motivational sequence, as specified in H2-H5 (based on the differential patterns suggested by Tylka & Homan, 2015; Salvador et al., 2023).*

**H2.**  *Body image satisfaction will be positively associated with perceived need-supportive behaviors from exercise professionals, whereas overweight dissatisfaction will be positively associated with perceived need-thwarting behaviors (Ntoumanis et al., 2017; Rodrigues et al., 2021b; Rocchi et al., 2017).*

**H3.**  *Perceived need-supportive behaviors will be positively associated with need satisfaction, while perceived need-thwarting behaviors will be positively associated with need frustration (Deci et al., 2017; Rodrigues et al., 2020a, 2020b).*

**H4.**  *Need satisfaction will be positively associated with more self-determined motivation, whereas need frustration will be positively associated with more non-self-determined motivation (Ryan & Deci, 2000; Panão & Carraça, 2020).*

**H5.**  *Self-determined motivation will be positively associated with intention for exercise persistence, while non-self-determined motivation will be negatively associated with it (Rodrigues et al., 2018; Rhodes & Kates, 2015).*

**H6.**  *The relationship between body image perception and intention for exercise persistence will be sequentially mediated by the distinct pathways outlined in H2 through H5 (i.e., perception of interpersonal behaviors → need satisfaction/frustration → motivational regulation), consistent with the integrated SDT process model (Ryan & Deci, 2000; Patrick & Williams, 2012).*

**H7.**  *It is hypothesized that the proposed structural model—including the pathways linking the perception of interpersonal behaviors, the satisfaction/frustration of basic psychological needs, the types of motivational regulation, and the outcome of exercise persistence intention—will demonstrate invariance between the body image satisfaction and dissatisfaction groups. This hypothesis is based on the core proposition of Self-Determination Theory that this integrated motivational sequence, from social context to behavioral intention, represents a fundamental and universal psychological process. Confirmation of this structural invariance would validate that any differences in the final intention to persist between groups are better explained by variations in the intensity of experiences along this universal pathway, and not by fundamentally different psychological mechanisms (Rodrigues et al., 2020a, 2020b; Ryan & Deci, 2017).*

### 1.6. Aim and Contribution

By testing this model, this study aims to provide a nuanced understanding of the motivational pathways through which body image influences exercise behavior. The findings will offer evidence-based insights for exercise professionals, highlighting how their interpersonal approach can be crucial in supporting clients, especially those struggling with body image, to foster sustainable exercise engagement.

## 2. Materials and Methods

Data collection took place after the study received approval from the ethics committee of the University of Extremadura (n. º 61/2022). There was a communication with gym-goers and exercise professionals, in which the study objectives were explained, and the principle of confidentiality was assured. Individuals invited to participate in this study either signed an informed consent form or approved it through an online method. After this step, questionnaires were distributed to exercise participants in that gym, both in online and paper formats.

### 2.1. Participants

This sample consisted of 821 participants aged between 18 and 65 years old (34.08 ± 11.92), with a total of 473 female participants and 348 male participants. This study comprised a sample of 821 participants from Portugal, aged between 18 and 65 years old (M = 34.08, SD = 11.92), of which 57.6% (n = 473) were female and 42.4% (n = 348) were male. Geographically, the sample shows a strong concentration in the central region of the country. The district of Leiria alone accounts for 59.7% (n = 490) of all respondents. Significant representation also comes from the districts of Santarém (13.6%, n = 112) and Setúbal (12.3%, n = 101). Other districts are represented in much smaller proportions, including Lisboa (3.3%, n = 27), Porto (1.3%, n = 11), and Coimbra (2.3%, n = 19), with several others having minimal representation (each ≤ 0.7%). This distribution indicates a notable regional focus that should be considered when interpreting the results. Regarding education, the sample is highly educated. The largest group holds a Bachelor’s degree, constituting 45.3% (n = 372) of participants. This is followed by those with Secondary education at 34.1% (n = 280). Furthermore, 15.0% (n = 123) hold a Master’s degree and 2.2% (n = 18) a Doctorate. Only 3.4% (n = 28) reported a basic education level. Concerning fitness habits, participants reported an average weekly training frequency of 3.70 times (SD = 1.29), ranging from 1 to 7 sessions. The average duration of their continuous practice was 32.56 months (SD = 48.76), though with high variability, ranging from 6 to 432 months. Additionally, for the purpose of present study two groups were created: 276 participants in the “Satisfied with Body Image” group with an average age of 33.22 ± 12.32 years, and 545 participants in the “Dissatisfied due to being overweight” group with an average age of 34.52 ± 11.69 years.

Participants had the sole requirement of engaging in physical exercise at a gym for a minimum of 6 months. They participated in activities such as personal training (PT), autonomous gym workouts, and group classes, with a frequency ranging from once a week to seven times a week.

The required sample size was calculated using Daniel Sopper’s online calculator (30), considering the following parameters: anticipated effect size (0.3), statistical power level (0.95), number of latent variables (7), and number of observed variables (21) (Westland, 2010). As a result, the minimum sample size needed to detect an effect was 247, while the minimum sample size required for the model structure was 200. The recommended minimum sample size was 247, which was adhered to in this study for the model.

### 2.2. Instruments

Interpersonal Behavior Questionnaire (IBQ)—Portuguese version (Rodrigues et al., 2021a). This questionnaire consists of 24 items, to which participants respond on a Likert scale ranging from 1 (completely disagree) to 7 (completely agree). Subsequently, the items are grouped into six factors: (a) autonomy support; (b) competence support; (c) relatedness support; (d) autonomy thwarting; (e) competence thwarting; (f) relatedness thwarting; underlying the SDT (Ryan & Deci, 2000). Recent studies, both in the general context (Rocchi et al., 2017) and in the sports context (Jacinto et al., 2024), have shown good data fit, with good adjusted internal consistency values, as well as good convergent and discriminant validity. In the present study, this questionnaire demonstrated the following internal consistency values: autonomy support (α = 0.84), competence support (α = 0.81), relatedness support (α = 0.88), autonomy thwarting (α = 0.82), competence thwarting (α = 0.85), and relatedness thwarting (α = 0.87). Furthermore, the confirmatory factor analysis indicated an acceptable fit to the data: χ^2^ = 359.23, B-S *p* < 0.001, CFI = 0.93, TLI = 0.91, SRMR = 0.06, RMSEA = 0.04 (90% CI [0.032, 0.087]).

Basic Psychological Needs Satisfaction and Frustration-Exercise (BPNSF-E)—Portuguese version (Rodrigues et al., 2019a). This questionnaire consists of 24 items, to which participants respond on a Likert scale ranging from 1 (not at all true for me) to 7 (totally true for me). Subsequently, the items are grouped into six factors: (a) autonomy satisfaction; (b) competence satisfaction; (c) relatedness satisfaction; (d) autonomy frustration; (e) competence frustration; (f) relatedness frustration; underlying the SDT (Ryan & Deci, 2017). Two composite factors (one reflecting basic psychological needs satisfaction and the other reflecting basic psychological needs frustration) were created, considering the study’s objective as recommended in several studies (Rodrigues et al., 2020b). Recent studies in several countries (Chen et al., 2015; Monteiro et al., 2025) have demonstrated that the measurement model showed good fit values, good internal consistency, and convergent and discriminant validity. The questionnaire used in this study showed good reliability, with the following Cronbach’s alpha coefficients for its subscales: autonomy satisfaction (α = 0.77), competence satisfaction (α = 0.86), relatedness satisfaction (α = 0.87), autonomy frustration (α = 0.81), competence frustration (α = 0.80), and relatedness frustration (α = 0.87). The instrument’s factor structure was validated through confirmatory factor analysis, which yielded an acceptable model fit: χ^2^ = 498.34, B-Sp < 0.001, CFI = 0.94, TLI = 0.93, SRMR = 0.05, RMSEA = 0.06, 90% CI [0.046, 0.067].

Behavioral Regulation Exercise Questionnaire (BREQ-3)—Portuguese version (Cid et al., 2018). This questionnaire is a self-report instrument developed by Markland and Tobin (2004), translated and validated for the Portuguese language for the context of physical exercise in gyms and fitness centers (Cid et al., 2018). The questionnaire consists of 24 items designed to assess the type of motivational regulation related to exercise participation. Participants respond on a Likert scale with 5 response levels, ranging from 0 (completely disagree) to 4 (completely agree). Subsequently, the items are grouped into six factors: amotivation, external regulation, introjected regulation, identified regulation, integrated regulation, and intrinsic motivation, underlying the motivational continuum of SDT (Ryan & Deci, 2000). For the purpose of the present study, the items were grouped into two composite factors: self-determined motivation (including identified and integrated regulation and intrinsic motivation) and non-self-determined motivation (amotivation, external and introjected regulation), as recommended by other studies (Rodrigues et al., 2020b). Recent studies have demonstrated the suitability of this questionnaire in the exercise context (Cid et al., 2018). The questionnaire demonstrated strong internal consistency across all motivational regulation subscales, with Cronbach’s alpha values ranging from 0.79 to 0.91: amotivation (α = 0.88), external regulation (α = 0.81), introjected regulation (α = 0.79), identified regulation (α = 0.80), integrated regulation (α = 0.85), and intrinsic motivation (α = 0.91). Confirmatory factor analysis supported the instrument’s proposed factor structure, with the model showing acceptable fit to the data: χ^2^ = 657.88, B-S *p* < 0.001, CFI = 0.92, TLI = 0.91, SRMR = 0.06, RMSEA = 0.07, 90% CI [0.058, 0.092].

Intention to continue exercising—Ajzen (2006) recommendations were followed, and three specific items were created to evaluate intention to continue exercising in the next six months: “I will continue to exercise in the next few months as I currently do or in a very similar way (same type, frequency, duration, intensity).” “I intend to continue practicing exercise in the next few months with the same dynamics or similar to what I do today (same type, frequency, duration, intensity).” and “I will continue to engage in physical exercise in the next few months as I currently do or in a very similar way (same type, frequency, duration, intensity).” A 7-point Likert scale anchored from 1—“completely disagree” to 7—“completely agree” was used to respond to these items. The questionnaire demonstrated strong internal consistency for the intention scale (α = 0.96). Confirmatory factor analysis confirmed the instrument’s proposed single-factor structure, with the model showing excellent fit to the data: χ^2^ = 68.57, B-S *p* < 0.001, CFI = 0.97, TLI = 0.96, SRMR = 0.03, RMSEA = 0.04, 90% CI [0.032, 0.067].

Stunkard’s Figure Rating Scale, adapted for the Portuguese population (Scagliusi et al., 2006), is a scale used to assess self-perception of body image and ideal body sizes. Participants viewed a matrix of nine body sizes and were asked to assign a figure corresponding to their current body shape. They were then questioned about how they perceived their own shape and asked to assign a figure that matched their ideal body shape, indicating how they would like to appear. Each of the nine figures represents a number from 1 to 9, with 1 representing the leanest body and 9 representing the most voluminous body. Silhouette figures are widely used to assess body size, and their validity and reliability are considered appropriate (needs references). Body image satisfaction was evaluated by calculating the difference between the rating obtained for the question about how one sees oneself and the rating obtained for the question about how one would like to be seen. This difference can range from +8 to −8. An individual is considered satisfied with their appearance when this difference is equal to 0. If the difference is positive, the individual is dissatisfied with being overweight. On the other hand, if it is negative, there is dissatisfaction with thinness (Pereira et al., 2009).

### 2.3. Data Analysis

Means, standard deviation, and bivariate correlations were calculated for the studied variables. In addition, a two-step maximum likelihood (ml) approach based on Kline (2016) was performed through IBM SPSS AMOS v.26. First, a confirmatory factor analysis (CFA) was conducted to test the psychometric properties of purpose models. average variance extracted (AVE), was performed to analyze the convergent validity and scores ≥ 0.50 were defined as acceptable (Hair et al., 2019). Discriminant validity was also evaluated to demonstrate whether the constructs under examination are sufficiently distinct from one another. It was confirmed when the values of AVE were higher than the squared correlation across constructs of the measurement model (Byrne, 2016; Hair et al., 2019). Finally, internal consistency was measured via composite reliability (CR), considering scores ≥ 0.70 (43). Second, structural equation modeling (SEM) was conducted to examine the relationships among the studied variables. Both standardized direct and indirect associations on the outcome variable were analyzed, with coefficients considered significant if the 95% confidence intervals (CIs) did not include zero (Williams & MacKinnon, 2008). The significance of the direct and indirect associations was assessed using Bootstrap resampling (1000 samples) with a bias-corrected 95% CI. For both models (CFA and SEM), traditional incremental and absolute indexes were employed to evaluate model fit. The following cutoff values were applied: comparative fit index (CFI), Tucker–Lewis index (TLI), standard root mean residual (SRMR), root mean square error of approximation (RMSEA), and its 90% confidence interval (CI: 90%). The cutoff values used were based on recommendations from several authors (Byrne, 2016; Hair et al., 2019; Marsh et al., 2004): CFI and TLI ≥ 0.90, SRMR and RMSEA ≤ 0.08. A multigroup structural equation modeling (SEM) analysis was performed to test the invariance of the hypothesized model across key subgroups, specifically comparing satisfaction with weight and dissatisfaction from being overweight. The analysis followed Byrne’s (2016) procedure for assessing configural, metric, scalar, and residual invariance. It proceeded in two main stages: (1) establishing a well-fitting baseline model for each subgroup, and (2) systematically testing for invariance by comparing progressively constrained models. Constraints were sequentially applied to measurement weights (metric invariance), structural weights, measurement intercepts (scalar invariance), and residuals. Model fit was evaluated using standard indices. Following the recommendations of Cheung and Rensvold (2002), invariance at each step was considered tenable if the change in the comparative fit index (ΔCFI) between the constrained and unconstrained models was 0.01 or less, which is a more robust criterion than reliance on chi-square difference tests alone.

## 3. Results

An inspection of data revealed that no univariate or multivariate outliers were found. Item-level descriptive statistics indicated that no violation form of normal distribution was observed since the skewness ranged from −1.98 to +1.98; kurtosis ranged from −7 to +7. Nevertheless, Mardia’s coefficient for multivariate kurtosis exceeded expected values (>5). Therefore, Boostrapp Bollen–Stine (2000 samples) was performed for further analysis (Nevitt & Hancock, 2001). In addition, the collinearity diagnosis was checked via Variance Inflation Factor (VIF). The VIF results demonstrated scores below 10, demonstrating, thus, the appropriate conditions to test the regression model.

Descriptive statistics showed that the participants presented scores above midpoint for perceived supportive behaviors, basic psychological needs satisfaction, self-determined motivation and intention and scores below midpoint for thwarting supportive behaviors, basic psychological needs frustration and non-self-determined motivation. Participants from Model 1 reported higher scores on perceived supportive behaviors, basic psychological needs satisfaction, and self-determined motivation compared to participants from Model 2. In addition, participants from Model 2 exhibited higher scores on thwarting supportive behaviors, basic psychological needs frustration and non-self-determined compared to participants from Model 1 as seen in Table 1. The bivariate correlations patterns showed a positive and significant associations across perceived supportive behaviors, basic psychological needs satisfaction, self-determined motivation, and intention to continue exercise. Additionally, the negative and significant associations were observed across thwarting supportive behaviors, basic psychological needs frustration, non-self-determined motivation and intention to continue exercise. These associations were consistent between two models (i.e., Model 1: satisfied with their weight; Model 2: dissatisfaction from being overweight).

The measurement model, comprising the factors of perceived supportive and thwarting behaviors, basic psychological needs satisfaction and frustration, self-determined and non-self-determined motivation, and intention to continue exercise, demonstrated adequate fit to the data in both models (see Table 2). For Model 1, the confirmatory factor analysis showed the following: χ^2^(168) = 564.85, χ^2^/df = 3.36, CFI = 0.942, TLI = 0.931, SRMR = 0.072, RMSEA = 0.078 (90% CI: 0.069–0.086). For Model 2, the results were as follows: χ^2^(168) = 976.72, χ^2^/df = 5.81, CFI = 0.931, TLI = 0.922, SRMR = 0.065, RMSEA = 0.074 (90% CI: 0.061–0.084). The composite reliability (CR) coefficients for each factor exceeded the cutoff value (>0.70), indicating good internal consistency. Based on the measurement model results and reliability analysis, both convergent and discriminant validity were assessed in each sample. Convergent validity was confirmed, as average variance extracted (AVE) scores were above the acceptable threshold, as shown in Table 1. Discriminant validity was also supported, as the squared correlations for each latent variable were lower than the corresponding AVE scores. These results provide preliminary support for conducting SEM analysis in each sample to examine the direct and indirect associations among the variables under study. The results from the SEM analysis showed that the structural model in each sample provided an acceptable fit to the data. For Model 1, the structural equation model showed the following: χ^2^(178) = 598.01, χ^2^/df = 3.35, CFI = 0.921, TLI = 0.911, SRMR = 0.078, RMSEA = 0.068 (90% CI: 0.048–0.086). For Model 2, the fit indices were as follows: χ^2^(178) = 668.99, χ^2^/df = 3.75, CFI = 0.924, TLI = 0.913, SRMR = 0.078, RMSEA = 0.063 (90% CI: 0.050–0.076).

**Figure 1:**
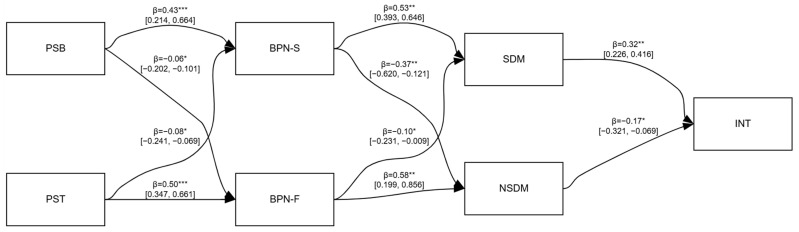
Model 1: satisfied with their weight. Note. PSB = perceived supportive behaviors; PST = perceived thwarting behaviors; BPN-S = basic psychological needs satisfaction; BPN-F = basic psychological needs frustration; SDM = self-determination motivation; NSDM = non-self-determined motivation; INT = intention to continue exercise; *** = significant path (*p* < 0.001); ** = significant path (*p* < 0.01); * = significant path (*p* < 0.05).

**Figure 2:**
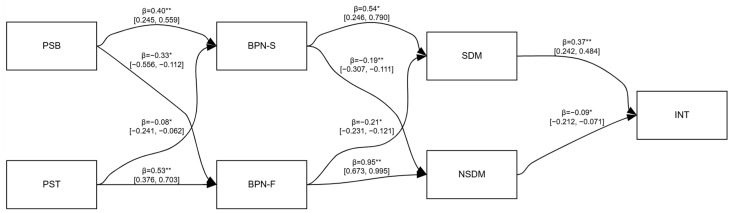
Model 2: Dissatisfaction from being overweight. Note. PSB = perceived supportive behaviors; PST = perceived thwarting behaviors; BPN-S = basic psychological needs satisfaction; BPN-F = basic psychological needs frustration; SDM = self-determination motivation; NSDM = non-self-determined motivation; INT = intention to continue exercise; ** = significant path (*p* < 0.01); * = significant path (*p* < 0.05).

The structural equation modeling results, illustrated in Figure 1 and Figure 2 and detailed in Table 3, confirmed the hypothesized model across both samples. The pattern of direct associations revealed two distinct motivational pathways. On one hand, perceived supportive behaviors (PSBs) were strongly positively associated with basic psychological needs satisfaction (BPN-S), with standardized coefficients of 0.43 and 0.40 in Model 1 and 2, respectively (*p* < 0.01). This satisfaction, in turn, showed a strong positive association with self-determined motivation (SDM; β = 0.53 and 0.54). On the other hand, perceived thwarting behaviors (PTBs) were strongly linked to basic psychological needs frustration (BPN-F with a strong association in Model 2 (β = 0.95, *p* = 0.002). BPN-F was then strongly and positively associated with non-self-determined motivation (NSDM). Finally, the direct paths to exercise intention (INT) were significant and opposing: SDM showed a consistent positive association (β = 0.32 and 0.37), while NSDM was negatively associated with it (β = −0.17 and −0.09).

The analysis of indirect associations revealed significant pathways. Perceived supportive behaviors demonstrated a positive indirect association with self-determined motivation through basic psychological needs satisfaction, and with intention to continue exercising through the sequential pathway of needs satisfaction and self-determined motivation. Additionally, perceived supportive behaviors showed a negative indirect association with non-self-determined motivation via basic psychological needs frustration. Conversely, perceived thwarting behaviors exhibited a significant negative indirect association with both self-determined motivation and exercise intention through basic psychological needs frustration and the subsequent motivational pathway. Furthermore, perceived thwarting behaviors demonstrated a positive indirect association with non-self-determined motivation through basic psychological needs frustration. Finally, basic psychological needs satisfaction showed a positive indirect association with exercise intention through self-determined motivation, while needs frustration demonstrated a negative indirect association with intention through non-self-determined motivation. These pathways were consistent across both models, as detailed in Table 3. The results of the sequential multigroup invariance testing are presented in Table 4. The unconstrained model (UM) demonstrated an acceptable baseline fit (CFI = 0.912). The imposition of equality constraints revealed the degree of model invariance across groups (i.e., satisfied with their weight vs. dissatisfaction with being overweight). Invariance was supported for the measurement weights (MW; ΔCFI = 0.002), structural weights (SM; ΔCFI = 0.003), structural covariances (SC; ΔCFI = 0.005), and structural residuals (SR; ΔCFI = 0.008), as the change in CFI (ΔCFI) for each step remained below the recommended cutoff of 0.01 (Cheung & Rensvold, 2002). However, the model constraining the measurement residuals (MRs) yielded a ΔCFI of 0.010, indicating a potential lack of strict invariance at this level, although the absolute CFI value (0.902) remained acceptable.

## 4. Discussion

The present study tested the integrated hypothesis that satisfaction versus dissatisfaction with body image (due to being overweight) is associated with distinct, yet psychometrically comparable, motivational pathways within the SDT framework. The results provide strong and consistent support for this hypothesis, revealing a clear divergence in the motivational profiles of individuals based on their body image perceptions.

The results reveal a pronounced distinction between the two groups. Participants satisfied with their body image reported significantly higher levels of perceived need-supportive behaviors from instructors, greater satisfaction of autonomy, competence, and relatedness, and more self-determined forms of motivation. In contrast, those dissatisfied due to being overweight reported higher levels of perceived need-thwarting behaviors, greater BPN frustration, and higher levels of non-self-determined motivation. This clear dichotomy is not a set of independent findings but represents the core empirical evidence for the distinct motivational pathways central to our hypothesis. This pattern aligns with previous research (Rodrigues et al., 2020a, 2020b; Salvador et al., 2023), indicating that body image is a core psychological factor associated with distinct motivational profiles among exercisers. One potential explanation for this divergence lies in cognitive-affective processing (Wang et al., 2025). Individuals with body dissatisfaction may enter exercise settings with a heightened sensitivity to evaluative cues (Puhl & Heuer, 2010). This disposition is associated with a tendency to perceive ambiguous instructor feedback as critical, which in turn correlates with reports of need frustration and non-self-determined motivation.

Delving deeper into these pathways, the structural model reveals that for body-satisfied individuals, a strongly positive motivational pattern emerges. Their perception of instructors’ need-supportive behaviors—such as providing meaningful rationales (autonomy support), offering constructive and positive feedback (competence support), and demonstrating genuine care and connection (relatedness support)—was significantly and positively associated with BPN satisfaction. This finding resonates strongly with the work of Van den Berghe et al. (2013), who highlighted the importance of perceived support from authority figures across contexts. This satisfaction of BPN was strongly and positively associated with more self-determined motivation (Rodrigues et al., 2020a, 2020b). According to SDT, when individuals experience feelings of volition, effectiveness, and connection, they tend to report greater internalization of the value of exercise and engagement driven by inherent enjoyment or personal significance (Ryan & Deci, 2017). In our study, this process was reflected in the finding that self-determined motivation showed a direct and strong positive association with a greater intention to continue exercising, which aligns with recent findings by Rodrigues et al. (2020a, 2020b) in the exercise context. Taken together, these associations form a coherent adaptive pathway—from supportive context to need satisfaction, to self-determined motivation, and finally to stronger persistence intentions—which align with and provides detailed evidence for one half of our hypothesized model. This entire observed pathway is aligned with the core propositions of SDT and suggests a potential route to long-term adherence for a significant portion of the exercising population (Ntoumanis & Standage, 2009; Rodrigues et al., 2018).

Conversely, for individuals dissatisfied due to being overweight, an equally distinct but less adaptive pattern was identified. This pattern constitutes the second, maladaptive pathway proposed in our hypothesis. In this group, the perception of need-thwarting behaviors—such as excessive control, critical feedback, or a lack of personal connection—was strongly associated with the frustration of their basic psychological needs. According to SDT, this experience of need frustration is theorized to be linked to maladaptive motivational styles (Ntoumanis et al., 2017). Specifically, BPN frustration was strongly and positively associated with non-self-determined motivation, including external regulation (e.g., exercising for rewards or approval) and introjected regulation (e.g., exercising to avoid guilt or shame). This type of motivation is typically fragile and correlates with negative emotional experiences, higher perceived exertion, and eventual behavioral dropout (Rodrigues et al., 2018; Teixeira et al., 2012). The significant negative association between non-self-determined motivation and the intention to continue exercising is a central finding for this group and provides critical evidence for the detrimental endpoint of the maladaptive pathway. One possible interpretation is a self-reinforcing cycle: the experience of a frustrating social environment in the gym may align with pre-existing body image concerns, which is in turn associated with a pressured and unpleasant form of motivation. This motivational profile is then linked to a higher likelihood of discontinuation (Ntoumanis et al., 2017). Consequently, individuals reporting this pattern of experiences might be less likely to access the health and well-being benefits that are often associated with the development of a more positive body image over time (Salvador et al., 2023).

The meaningful comparison of these motivational pathways is psychometrically supported by the confirmation of structural invariance for the hypothesized model. The analysis established all types of invariances meeting the recommended thresholds for group comparisons in latent variable modeling (Cheung & Rensvold, 2002). This indicates that the constructs of perceived interpersonal behaviors, basic psychological needs, and motivational regulations were measured equivalently across both groups. This methodological rigor is essential, as it allows us to confidently interpret the divergent pathways not as measurement artifacts, but as genuine psychological differences associated with body image perception, thereby strengthening the evidence for our hypothesis. Specifically, the same questionnaire items loaded onto their respective latent factors with statistically equivalent strength in both groups, and the baseline levels of these items were comparable. Therefore, the observed divergences in the structural model can be confidently interpreted as genuine differences in psychological experiences and processes, rather than artifacts of measurement bias or differential item interpretation (Byrne, 2016). This comprehensive level of invariance aligns with rigorous methodological standards in SDT research (Rodrigues et al., 2021a) and substantiates the premise that body image perception delineates distinct, yet psychometrically comparable, motivational profiles. In other words, the “rules” of the motivational sequence are the same, but the starting point of body image significantly alters how the sequence unfolds and its ultimate outcome regarding persistence intentions (Ryan & Deci, 2017; Tylka & Homan, 2015).

The clear distinction observed between the two motivational pathways highlights a consistent association with the perceived interpersonal style of exercise professionals. Their role extends beyond technical expertise; they can act as key facilitators whose perceived behavior shows a strong association with a client’s motivational experience (Rodrigues et al., 2018, 2020a, 2020b). This association may be especially pertinent for clients with body image dissatisfaction, who might be particularly sensitive to the perceived social climate of a gym environment (Puhl & Heuer, 2010). For these individuals, a generic approach may be insufficient. Instead, a deliberate, person-centered, and need-supportive approach is often recommended in the SDT literature, which could involve the following: fostering autonomy by offering meaningful choices and acknowledging feelings without judgment (Ryan & Deci, 2000; Patrick & Williams, 2012); building competence by providing feedback focused on effort, technique, and personal improvement rather than on appearance (Van den Berghe et al., 2013; Rodrigues et al., 2021a, 2021b); and enhancing relatedness by demonstrating empathy and fostering a sense of belonging (Ntoumanis et al., 2017; Rodrigues et al., 2020a, 2020b). The intentional use of such strategies could help clients reframe the experience of exercise, separating it from external pressures and body-related anxieties (Panão & Carraça, 2020; Tylka & Homan, 2015). This may support a reframing of physical activity as an opportunity for self-care, mastery, and positive social connection, which is theoretically and empirically linked to more self-determined motivation (Rodrigues et al., 2018; Teixeira et al., 2012). Adopting such a supportive approach is aligned with SDT principles and could be conducive to greater exercise adherence and potentially contribute to overall psychological well-being (Hagger & Chatzisarantis, 2008; Edmunds et al., 2008; Jankauskiene et al., 2026).

## 5. Practical Implications

Aligned with the Self-Determination Theory (SDT) framework, this study examined the relationships between body image perception and key motivational constructs. The confirmation of two distinct pathways offers clear, integrated insights for practice. The observed associative patterns offer insights for exercise and health professionals, suggesting that interpersonal behaviors perceived as supportive of autonomy, competence, and relatedness are correlated with more adaptive motivational profiles and a stronger intent to maintain physical activity (Rodrigues et al., 2020a, 2020b). These findings underscore a potential link between professional conduct and client motivation. Beyond the fitness context, the observed associations could inform health promotion and clinical practice. Understanding these motivational correlations may aid healthcare providers in designing more patient-centered interventions (Patrick & Williams, 2012). Integrating principles from SDT into healthcare could provide a framework to support patients’ self-determined motivation, which has been associated with better adherence to health behaviors in prior research (Ntoumanis et al., 2021; Teixeira et al., 2012; Jankauskiene et al., 2026).

From a public health and clinical perspective, fostering environments that align with SDT principles is a strategy consistent with efforts to enhance engagement in physical activity (Guthold et al., 2018, 2020). Such environments could potentially contribute to broader public health goals, including the management of chronic conditions (Lee et al., 2012; Wang et al., 2025). Furthermore, creating supportive, non-judgmental settings may help address some of the adverse psychological correlations of body dissatisfaction and promote well-being (Puhl & Heuer, 2010; Van den Berghe et al., 2013). Encouraging autonomy-supportive communication styles could also be a relevant consideration for reducing perceived weight-related stigma and strengthening therapeutic alliances (Puhl & Heuer, 2010). In summary, these findings lend support to a motivationally informed, patient-centered perspective in healthcare (Jankauskiene et al., 2026). By considering the role of perceived need-supportive interactions and self-determined motivation, healthcare professionals may find value in developing interventions informed by these principles (Hagger & Chatzisarantis, 2008). This approach aligns with global health priorities emphasizing prevention, self-management, and empowerment (Bull et al., 2020).

## 6. Limitations and Conclusions

### 6.1. Limitations and Future Research Directions

This study provides important insights but must be interpreted within the context of its limitations, which also charts a course for future inquiry.

The cross-sectional design precludes definitive causal conclusions. While the hypothesized pathways are grounded in Self-Determination Theory, longitudinal or experimental studies are needed to verify causal direction and temporal dynamics, such as how fluctuations in body image influence motivational processes over time.

Generalizability is constrained by the sample of gym-goers, excluding individuals from other exercise contexts (e.g., home, outdoor, sports clubs). Future research should test the model in more diverse settings. The operationalization of body image as a dichotomous group based on the Stunkard scale difference score, while justified, results in a loss of information regarding the intensity of dissatisfaction and excludes individuals with “lean dissatisfaction”. Thus, findings are specific to the contrast with overweight-related dissatisfaction.

The reliance on self-report measures introduces potential biases like social desirability and common method variance. Key behavioral variables, such as detailed exercise volume and modality, were not specified or controlled for, which could confound motivational differences. Future studies would benefit from objective behavioral data (e.g., accelerometers, attendance records). Furthermore, interpersonal behaviors were assessed solely through participant perception, which captures the psychologically salient experience but not the instructors’ objective behaviors.

Finally, while measured, demographic variables like gender and age were not tested as moderators within the model, as the analytical focus was on the core pathways between body image groups. This limits generalizability across subgroups. Future studies should investigate these and other contextual moderators, such as whether the exercise environment promotes an “idealized body” imagery, to enable more tailored intervention strategies.

### 6.2. Conclusions

Despite these limitations, the study yields robust and clear conclusions. The establishment of measurement invariance confirms that the constructs were measured equivalently across groups, allowing for a confident interpretation of the distinct motivational pathways observed.

The results reveal a systematic divergence in motivational experiences based on body image perception. Individuals satisfied with their body image tended to perceive their exercise instructors as more need-supportive. This perception was associated with greater satisfaction of their basic psychological needs for autonomy, competence, and relatedness, which in turn fostered more self-determined motivation and a stronger intention to persist with exercise. This pattern represents the adaptive, health-promoting motivational sequence outlined by Self-Determination Theory.

Conversely, individuals dissatisfied due to being overweight reported a markedly different pathway. They perceived more need-thwarting behaviors from instructors, which was associated with higher frustration of their basic psychological needs. This state of need frustration was linked to less self-determined motivation and, ultimately, a weaker intention for long-term exercise persistence.

In conclusion, this study successfully integrates body image as a key antecedent within the SDT framework, demonstrating that it is associated with individuals embarking on divergent motivational journeys through the exercise environment. Crucially, the model’s structural consistency across groups suggests that the core psychological process—from social perception to motivation—is universal. This reinforces the broad relevance of need-supportive principles. The findings strongly indicate that instructional approaches deliberately designed to foster autonomy, competence, and relatedness are vital. Such approaches are not only associated with better motivational outcomes but may be particularly crucial for supporting exercisers struggling with body image dissatisfaction, helping to steer them away from a frustrating pathway and towards a more sustainable and self-determined form of exercise engagement.

## Figures and Tables

**Table 1:** Descriptive statistics, bivariate correlations, average variance extracted values, and composite reliability coefficients.

Variables	Range	*M*	*SD*	1	2	3	4	5	6	7	AVE	CR
Model 1												
1. PSB	1–7	5.68	1.15	1	-	-	-	-	-	-	0.80	0.82
2. PST	1–6	2.02	1.04	−0.50 **	1	-	-	-	-	-	0.71	0.71
3. BPN-S	1–5	4.20	0.68	0.43 **	−0.25 **	1	-	**-**	**-**	-	0.71	0.78
4. BPN-F	1–5	1.64	0.67	−0.26 **	0.41 **	−0.48 **	1	-	-	-	0.76	0.80
5. SDM	1–4	3.44	0.56	0.34 **	−0.35 **	−0.09 *	0.40 **	1	-	-	0.77	0.81
6. NSDM	0–3	0.84	0.57	−0.17 **	0.26 **	0.45 **	−0.45 **	−0.18 **	1	-	0.61	0.70
7. INT	2–5	4.38	0.60	0.18 **	−0.13 **	0.16 **	−0.17 **	−0.12 *	0.20 **	1	0.92	0.92
Model 2												
1. PSB	2–7	5.60	1.08	1	-	-	-	-	-	-	0.80	0.81
2. PST	1–6	2.04	0.87	−0.46 **	1	-	-	-	-	-	0.72	0.73
3. BPN-S	2–5	4.09	0.56	0.39 **	−0.26 **	1	-	**-**	**-**	**-**	0.71	0.77
4. BPN-F	1–5	1.79	0.63	−0.28 **	0.38 **	−0.59 **	1	-	-	-	0.76	0.80
5. SDM	0–4	3.27	0.57	0.29 **	−0.21 **	0.50 *	−0.35 **	1	-	-	0.75	0.80
6. NSDM	0–4	1.09	0.55	−0.14 **	0.32 **	−0.26 **	0.52 **	−0.17 **	1	-	0.62	0.71
7. INT	2–5	4.37	0.63	0.21 **	−0.21 **	0.10 **	−0.11 **	0.33 *	−0.16 **	1	0.93	0.93

**Note.** PSB = perceived supportive behaviors; PST = perceived thwarting behaviors; BPN-S = basic psychological needs satisfaction; BPN-F = basic psychological needs frustration; SDM = self-determination motivation; NSDM = non-self-determined motivation; INT = intention to continue exercise; Model 1: satisfied with their weigh; Model 2: dissatisfaction from being overweight; * *p* < 0.05; ** *p* < 0.01.

**Table 2:** Goodness-of-fit indexes for models analyzed.

Models	χ^2^	df	χ^2^/df	B-Sp	CFI	TLI	SRMR	RMSEA	90% CI
CFA–Model 1	564.85	168	3.36	0.001	0.942	0.931	0.072	0.078	0.069–0.086
CFA–Model 2	976.72	168	5.81	0.001	0.931	0.922	0.065	0.074	0.061–0.084
SEM–Model 1	598.01	178	3.35	<0.001	0.921	0.911	0.078	0.068	0.048–0.086
SEM–Model 2	668.99	178	3.75	<0.001	0.924	0.913	0.078	0.063	0.050–0.076

**Note.** CFA = confirmatory factor analysis; SEM = structural equation modeling; χ^2^ = chi-square; df = degrees of freedom; χ^2^/df = normalized chi-square; B-Sp = Bollen–Stine level of significance; CFI = comparative fit index; TLI = Tucker–Lewis index; SRMR = standardized root mean square residual; RMSEA = root mean square error of approximation; 90% CI = confidence interval at 90% for RMSEA; Model 1: satisfied with their weigh; Model 2: dissatisfaction from being overweight.

**Table 3:** Direct and indirect regression paths.

Regression Path	Direct				Indirect		
	β	95% CI	*p*		β	95% CI	*p*
Model 1				Model 1			
PSB → BPN-S	0.43	0.214, 0.664	0.007	PSB → SDM	0.24	0.145–0.358	0.008
PSB → BPN-F	−0.06	−0.202, −0.101	0.040	PSB → NSDM	−0.09	−0.187, −0.041	0.028
PST → BPN-S	−0.08	−0.241, −0.069	0.038	PSB → INT	0.10	0.047, −0.117	0.003
PST → BPN-F	0.50	0.347, 0.661	0.001	PST → SDM	−0.19	−0.379, −0.111	0.022
BPN-S → SDM	0.53	0.393, 0.646	0.002	PST → NSDM	0.25	0.011, 0.579	0.012
BPN-S → NSDM	−0.37	−0.620, −0.121	0.003	PST → INT	−0.08	−0.175, −0.039	0.033
BPN-F → SDM	−0.10	−0.231, −0.009	0.032	BPN-S → INT	0.17	0.114, 0.234	0.018
BPN-F → NSDM	0.58	0.199, 0.856	0.004	BPN-F → INT	−0.10	−0.206, −0.010	0.034
SDM → INT	0.32	0.226, 0.416	0.007	-	-	-	-
NSDM → INT	−0.17	−0.321, −0.069	0.036	-	-	-	-
Model 2				Model 2			
PSB → BPN-S	0.40	0.245, 0.559	0.003	PSB → SDM	0.24	0.116–0.318	0.018
PSB → BPN-F	−0.33	−0.556, −0.112	0.027	PSB → NSDM	−0.14	−0.112, −0.049	0.026
PST → BPN-S	−0.08	−0.241, −0.062	0.041	PSB → INT	0.16	0.098–0.132	0.031
PST → BPN-F	0.53	0.376, 0.703	0.002	PST → SDM	−0.22	−0.379, −0.128	0.004
BPN-S → SDM	0.54	0.246, 0.790	0.021	PST → NSDM	0.28	0.117, 0.487	0.001
BPN-S → NSDM	−0.19	−0.307, −0.111	0.003	PST → INT	−0.11	−0.191, −0.069	0.019
BPN-F → SDM	−0.21	−0.231, −0.121	0.032	BPN-S → INT	0.16	0.101, 0.310	0.009
BPN-F → NSDM	0.95	0.673, 0.995	0.002	BPN-F → INT	−0.18	−0.112, −0.298	0.010
SDM → INT	0.37	0.242, 0.484	0.003	-	-	-	
NSDM → INT	−0.09	−0.212, −0.071	0.036	-	-	-	

**Note.** PSB = perceived supportive behaviors; PST = perceived thwarting behaviors; BPN-S = basic psychological needs satisfaction; BPN-F = basic psychological needs frustration; SDM = self-determination motivation; NSDM = non-self-determined motivation; INT = intention to continue exercise; Model 1: satisfied with their weight; Model 2: Dissatisfaction from being overweight.

**Table 4:** Goodness-of-fit indexes for the invariance of the structural model across groups.

Model	χ^2^	df	∆χ^2^	∆df	*p*	CFI	∆CFI
UM	1893.789	356	-	-	-	0.912	-
MW	1934.249	370	40.459	14	<0.001	0.910	0.002
SM	1967.440	380	73.650	24	<0.001	0.909	0.003
SC	1982.892	383	89.102	27	<0.001	0.907	0.005
SR	1999.831	388	106.042	32	<0.001	0.904	0.008
MR	2063.900	409	170.111	53	<0.001	0.902	0.010

**Note.** χ^2^ = chi-square; ∆χ^2^ = differences in value of chi-square; ∆df = differences in degrees of freedom; *p* = level of significance; CFI = comparative fit index; ∆CFI = differences in the value of the comparative fit index; UM: unconstrained model; MW: measurement weights; SM: structural weights; SC: structural covariance’s; SR: structural residuals; MR: measurement residuals.

## Data Availability

The datasets used and/or analyzed during the current study are available from the corresponding author upon reasonable request.
